# P47 The conundrums of Macrophage activation syndrome (MAS) - when to consider a Haematopoietic Stem Cell Transplant (HSCT)

**DOI:** 10.1093/rap/rkac067.047

**Published:** 2022-09-28

**Authors:** Kathy Gallagher, Dearbhla McKenna, Charlene Foley, Ruqaiya Al Jashmi, Muthana Al Obaidi, Paul Brogan

**Affiliations:** Great Ormond Street Hospital for Children, London, United Kingdom; Great Ormond Street Hospital for Children, London, United Kingdom; Great Ormond Street Hospital for Children, London, United Kingdom; Great Ormond Street Hospital for Children, London, United Kingdom; Great Ormond Street Hospital for Children, London, United Kingdom; Great Ormond Street Hospital for Children, London, United Kingdom

## Abstract

**Introduction/Background:**

MAS is a severe, potentially fatal complication of systemic JIA (sJIA). It is characterised by uncontrolled activation and proliferation of T cells and macrophages, leading to a “cytokine storm” which manifests as hyperinflammation. Its consequences include fever, rash, cytopenias, transaminitis, coagulopathy and hyperferritinaemia.

Prompt careful assessment is required to identify MAS and exclude other conditions like sepsis. This is often done in parallel, performing a septic screen and commencing antibiotics whilst considering immunosuppressive therapy and its timing. MAS can be refractory to familiar first line agents, presenting a challenge for Paediatric Rheumatologists as no consensus guidelines exist to inform management.

**Description/Method:**

We present a case of a 15-month-old girl with sJIA and refractory MAS. Our patient became unwell at 8-months of age with fever and rash. She was initially treated with antibiotics. She then developed peeling of the fingers and toes. Management of Kawasaki Disease was initiated - IVIG (2 doses), IV methylprednisolone (IVMP) for 5 days, followed by oral prednisolone for ten days, and aspirin. Her fever persisted. The prednisolone and aspirin doses were increased which resulted in clinical improvement.

After a few weeks, the fever recurred three times daily and associated with an erythematous rash. She was admitted to hospital and treated with antibiotics for a presumed UTI. The diagnosis of sJIA was made after exclusion of other infectious and haematological causes. Despite IVMP followed by oral prednisolone and anakinra, the fever persisted.

She was transferred to our hospital for further management. Initial bloods showed Hb 103g/L, platelets 257x109/L, WBC 13.07x109/L, Neutrophils 4.18x109/L, ESR 32mm/hr, CRP 30mg/L, ferritin 2201ng/mL, ALT 89U/L, AST 218U/L, LDH 1859IU/L, triglycerides 4.2mmol/L, fibrinogen 2.8mg/dL, d-dimers 4641ng/mL. She was pulsed with IVMP for 3 days. The anakinra dose increased. Remission was not achieved biochemically. A further pulse with IVMP (3 days) was given with no improvement. IV ciclosporin was added. Tocilizumab 12mg/kg/day once daily was commenced. She remained on oral prednisolone 2mg/kg/day. She then developed sepsis. She had a positive blood culture from her PICC line. Despite IV antibiotics, her clinical picture didn’t improve. She eventually developed septic shock requiring a fluid bolus and worsening MAS. The PICC line was removed. She also required amlodipine for hypertension.

There was no sign of MAS remission (table 1), and the decision was made to commence etoposide (initial 150mg/M2 for 6 doses; current dose 50mg/M2) whilst working our patient up for a HSCT eight months after first presentation.

**Discussion/Results:**

MAS is a severe complication of sJIA. MAS can progress very rapidly and if left untreated, it is associated with high morbidity and mortality. Abnormal investigations include cytopenia, coagulopathy and hyperferritinaemia. These distinctive features usually occur in the later stages of MAS. This can lead to a delay in the diagnosis of the condition, resulting in a worse outcome. Early recognition of MAS is key to improving morbidity and mortality.

The mainstay of treatment for MAS is glucocorticoids. However, as this patient highlights glucocorticoids alone are not often satisfactory in controlling the disease. Other treatments can be used, but there is a paucity of data from clinical trials as to what treatment should be used and in what order.

HSCT is a potentially curative option for patients with refractory sJIA. Some of these patients can achieve drug free remission, although not all. In some, disease remission is only transient. HSCT is associated with significant risks, in particular Graft versus Host Disease. The data on success of HSCT in sJIA is sparce – making it challenging for clinicians to counsel parents.

Currently this patient’s condition is being controlled with Etoposide whilst HSCT is awaited. Etoposide is a well-established therapy for Primary Hemophagocytic Lymphhistiocytosis (HLH). As clinicians we are continuing to learn more about its use in controlling sJIA. A recent article by Horne et al suggests that a moderate dose of etoposide may be beneficial in severe/refractory HLH. We are currently trialling this approach in our patient with the hope of decreasing the overall Etoposide dose, aiming to minimise toxicity whilst adequately controlling her disease.

Finally, it is vital to screen for primary HLH in those whose first rheumatological presentation is with MAS. An important differential is a genetic cause which may or may not be identifiable.

**Key learning points/Conclusion:**

Learning points:

Improved awareness of MAS is paramount to aid timely diagnosis and ensure prompt treatment in order to try and improve MAS associated morbidity and mortality.

Careful evaluation of serum ferritin in conjunction with other blood parameters is important and helpful in monitoring response to therapy.

It is vital to screen for Primary HLH in patients whose first rheumatological presentation is MAS.

There is a lack of evidence for treatment pathways in refractory sJIA and MAS.

Some children develop refractory MAS and HSCT needs to be seriously considered, but when should clinicians consider HSCT?

Many unanswered questions exist. It can be challenging as clinicians to know when to refer patients for HSCT and how best to manage these patients whilst awaiting HSCT. It would be useful to develop consensus guidelines to help answer some of the following questions:

When should a child be referred for HSCT?

How many drugs should be trialled prior to referral?

What is the definition of treatment failure?

Who should decide that the child should be referred for HSCT?

What treatment should be used to bridge to HSCT and at what dose?

**P47 Table 1:** MAS clinical activity – partial remission

VAS	2 cm	YES
WBC	9.23 x10 9/L	> LLN - YES
Platelets	344 x10 9/L	> LLN - YES
LDH	483 IU/L	<1.5 ULN - NO
ALT	13 U/L	< 1.5 ULN - YES
AST	72 U/L	<1.5 ULN - NO
Fibrinogen	2.2 g/L	> 1 - YES
Ferritin	287 ng/mL	Decreased by 80% from values at baseline or < 2000mg/ml - YES

*LLN – lower limit of normal, ULN upper limit of normal*

